# Supplementary material for: Cerebrospinal Fluid and Peripheral Blood Lymphomonocyte Single-Cell Transcriptomics in a Subject with Multiple Sclerosis Acutely Infected with HIV
Source: Int J Mol Sci. 2024 Sep 28;25(19):10459. doi: 10.3390/ijms251910459 (PMC11476486; doi:10.3390/ijms251910459)

Figure S1. Brain and cervical spine Magnetic Resonance Imaging (MRI) scan of the MS patient, acutely infected with HIV. Panels A-C: Sagittal T2-weighted MRI of the cervical spine showing demyelinating lesions at C2-C3 and C6-C7 levels. Panels D, E: Axial FLAIR images displaying sporadic demyelinating lesions, with the largest in the left cerebellar peduncle.

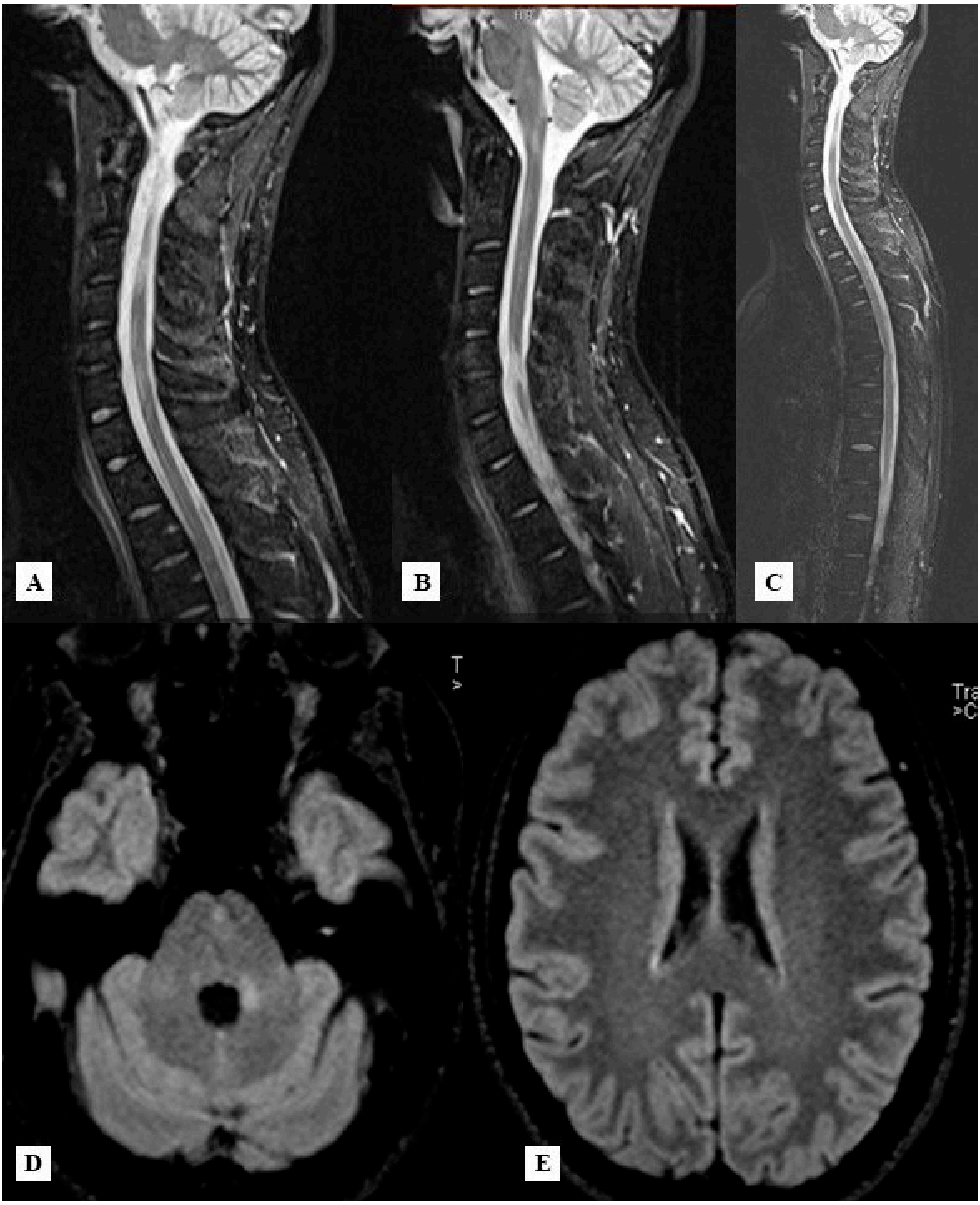

Supplement: Supplementary file 1 [file ijms-25-10459-s001.zip › Figure S1 Legend.pdf]
